# Supplementary material for: Complexome profiling of the Chlamydomonas psb28 mutant reveals TEF5 as an early PSII assembly factor
Source: Plant Cell. 2025 Mar 17;37(6):koaf055. doi: 10.1093/plcell/koaf055 (PMC12164590; doi:10.1093/plcell/koaf055)
Supplement: koaf055_Supplementary_Data [file koaf055_supplementary_data.zip › psb28_interactive.zip!/plots/intensity/qualityPlots/peptides/PHC43_Cre07.g330750.t1.1.html]

### Cre07.g330750.t1.1

Trivial name: PHC43  
SeqCoverage [median]: 1.80  
Mass: 95.32 kDa  
  
 

| Sequence | MissedCleavage | Mass [Da] | wtIntensity | muIntensity | Score |
| --- | --- | --- | --- | --- | --- |
| AAVVDATAK | 0 | 844.47 | 380.60 | 340.93 | 83.21 |
| ITALNANR | 0 | 871.49 | 382.14 | 568.83 | 84.24 |
|  |  |  | 762.7 | 909.8 |  |
